# Supplementary material for: Mechanisms governing the pioneering and redistribution capabilities of the non-classical pioneer PU.1
Source: Nat Commun. 2020 Jan 21;11:402. doi: 10.1038/s41467-019-13960-2 (PMC6972792; doi:10.1038/s41467-019-13960-2)
Supplement: Supplementary file 7 — Source data [file 41467_2019_13960_MOESM7_ESM.zip › Source_Data/FigureS2/FigureS2B_MotifScanOutput/KmeansCluster1/homerResults/motif7.similar.html]

motif7

## Information for motif7

G
A
C
T
A
G
T
C
G
A
T
C
G
A
C
T
A
G
T
C
G
A
T
C
G
A
C
T
A
G
T
C
G
A
T
C
G
A
C
T
A
G
T
C
G
A
T
C
G
A
C
T
A
G
T
C
  
Reverse Opposite:  

T
C
A
G
C
T
G
A
C
T
A
G
T
C
A
G
C
T
G
A
C
T
A
G
T
C
A
G
C
T
G
A
C
T
A
G
T
C
A
G
C
T
G
A
C
T
A
G
T
C
A
G
C
T
G
A
  

|  |  |
| --- | --- |
| p-value: | 1e-173 |
| log p-value: | -4.003e+02 |
| Information Content per bp: | 1.594 |
| Number of Target Sequences with motif | 3085.0 |
| Percentage of Target Sequences with motif | 60.60% |
| Number of Background Sequences with motif | 15767.4 |
| Percentage of Background Sequences with motif | 39.75% |
| Average Position of motif in Targets | 101.2 +/- 46.7bp |
| Average Position of motif in Background | 102.0 +/- 96.6bp |
| Strand Bias (log2 ratio + to - strand density) | 0.1 |
| Multiplicity (# of sites on avg that occur together) | 1.61 |
| Motif File: | file (matrix) reverse opposite |

### Similar de novo motifs found

|  |  |  |  |  |  |  |  |
| --- | --- | --- | --- | --- | --- | --- | --- |
| Rank | Match Score | Redundant Motif | P-value | log P-value | % of Targets | % of Background | Motif file |
| 1 | 0.838 | T G A C G T A C A G C T A G T C T G A C G C A T T A G C G T A C C A G T T G A C G T A C G C A T T A G C A G T C | 1e-69 | -160.616272 | 6.48% | 1.80% | motif file (matrix) |
